# Simulating digital micromirror devices for patterning coherent excitation light in structured illumination microscopy

**DOI:** 10.1098/rsta.2020.0147

**Published:** 2021-06-14

**Authors:** Mario Lachetta, Hauke Sandmeyer, Alice Sandmeyer, Jan Schulte am Esch, Thomas Huser, Marcel Müller

**Affiliations:** ^1^ Biomolecular Photonics, Faculty of Physics, Bielefeld University, Universitätsstraße 25, 33501 Bielefeld, Germany; ^2^ Numerical Simulations and Field Theory, Faculty of Physics, Bielefeld University, Universitätsstraße 25, 33501 Bielefeld, Germany; ^3^ Department of General and Visceral Surgery, Evangelisches Klinikum Bethel GmbH, University Hospital OWL of Bielefeld University, Campus Bielefeld-Bethel, Bielefeld, Germany

**Keywords:** structured illumination microscopy, digital micromirror device, coherent light simulation, super-resolution optical microscopy

## Abstract

Digital micromirror devices (DMDs) are spatial light modulators that employ the electro-mechanical movement of miniaturized mirrors to steer and thus modulate the light reflected off a mirror array. Their wide availability, low cost and high speed make them a popular choice both in consumer electronics such as video projectors, and scientific applications such as microscopy. High-end fluorescence microscopy systems typically employ laser light sources, which by their nature provide coherent excitation light. In super-resolution microscopy applications that use light modulation, most notably structured illumination microscopy (SIM), the coherent nature of the excitation light becomes a requirement to achieve optimal interference pattern contrast. The universal combination of DMDs and coherent light sources, especially when working with multiple different wavelengths, is unfortunately not straight forward. The substructure of the tilted micromirror array gives rise to a *blazed grating,* which has to be understood and which must be taken into account when designing a DMD-based illumination system. Here, we present a set of simulation frameworks that explore the use of DMDs in conjunction with coherent light sources, motivated by their application in SIM, but which are generalizable to other light patterning applications. This framework provides all the tools to explore and compute DMD-based diffraction effects and to simulate possible system alignment configurations computationally, which simplifies the system design process and provides guidance for setting up DMD-based microscopes.

This article is part of the Theo Murphy meeting ‘Super-resolution structured illumination microscopy (part 1)’.

## 1. Introduction

Spatial light modulators (SLMs) offer a robust and fast way to pattern the excitation light in a fluorescence microscope. This can be employed for various illumination schemes [1], for example to achieve selective (de)activation of photo-switchable dyes [2], and most notably, to achieve background suppression and resolution enhancement in structured illumination microscopy (SIM) [3–8], a widely used, fast super-resolution microscopy technique [9–13]*.* Typical SLMs are based on liquid crystal technology, and thus operate by electrically modulating the phase (or amplitude) of light through their active material. Digital micromirror devices (DMDs), on the other hand, work electro-mechanically, by individually flipping the orientation of each mirror between two pre-defined states. Because of their widespread use in consumer devices such as video projectors, DMDs are available at relatively low cost and in a variety of sizes. They also offer high switching speeds, they can handle high light intensities, and depending on coating, are not sensitive to light polarization. This makes them an interesting option for many SLM applications in microscopy [14–22]. However, the jagged nature of the micromirror array gives rise to the *blazed grating effect* that becomes rather annoying and detrimental when using DMDs in combination with a coherent light source [23,24]. Thus, if DMDs are selected as active light modulation systems in a fluorescence microscope based on laser light sources, this effect must be well understood and needs to be taken into consideration.

**Figure 1.**
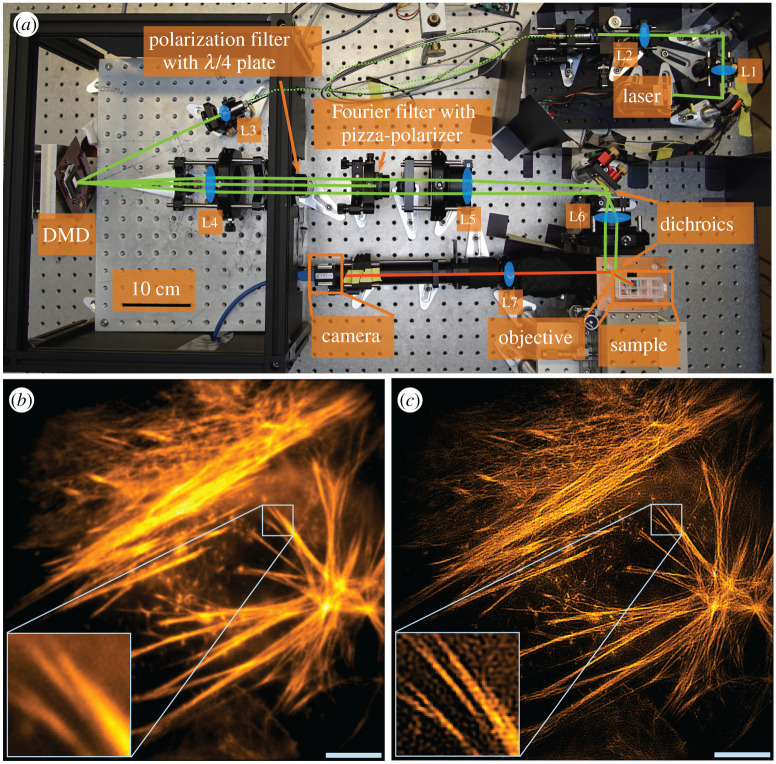
DMD-SIM microscope and measurements. (*a*) Compact and cost-effective SIM system based on a DMD with a 532 nm laser. The design of this microscope necessitated and motivated the research presented in this article. (*b*) 36 µm × 36 µm wide field of fixed U2OS cell labelled with Phalloidin Atto532 acquired using the instrument with 20 ms exposure per raw frame. The actin filaments are not distinguishable. (*c*) SIM data of (*b*). The actin filaments are distinguishable (scale bar 5 µm, inset 2.8 µm × 2.8 µm). (Online version in colour.)

The work presented here was motivated by our wish to expand the range of applications of a DMD-based SIM system (figure 1), where a coherent laser source is the primary source for the SIM interference pattern, giving rise to optimal pattern contrast [14]. We have developed a set of simulation frameworks that allow us to simulate the propagation of coherent light reflected off a DMD at different angles of incidence, at different wavelengths and with the DMD displaying arbitrary patterns. This enables us to explore the feasibility of optical layouts, to determine which simplifications (e.g. keeping optical elements in a single plane on a table) are possible, and to choose the proper wavelength combinations that could be used in a multi-colour DMD-based system. While our work is motivated by and centred around SIM, most of the findings should apply to any other, arbitrary DMD-based microscope systems that employ coherent illumination.

## 2. Methods

If DMDs are to be used with coherent illumination, it is essential to know and understand the resulting diffraction patterns generated by both the pattern displayed on the DMD and, much more importantly, the structure of the tilted micromirrors that is native to the device. Harvey & Pfisterer [25] provide a one-dimensional introduction to the blazed grating effect itself, but this model needs to be extended when used to describe a two-dimensional DMD with mirrors tilting along their diagonal. A white paper [23] by Texas Instruments (itself a large producer of DMD chips) offers an introduction to the blazed grating effect in conjunction with DMDs, and Brown *et al*. [22] recently provided a (semi-)analytic framework to model DMDs. Simultaneously, we started from a purely numerical approach to simulate light propagation in a DMD-based system, and developed simplifications to this model in the process. This results in three simulation algorithms, each with different assumptions and thus restrictions made to the system simulated, and thus different resulting strengths. Additionally, we have developed an analytical solution that can be used along the diagonal of the DMD, and is in agreement with the results found by Brown *et al*. All three algorithms are based on the same physical model of propagating electromagnetic fields interacting with the DMD's structure.

### (a) General modelling of coherent light and DMDs

The general model was derived for the previously developed DMD-based SIM system [14], and, for completeness, is summarized here, as it forms the basis of our simulation approaches. For incident and diffracted directions we use the normalized vectors $\vec{a}=(a_{x},a_{y},a_{z})$ and $\vec{b}=(b_{x},b_{y},b_{z})$ with the angle coordinates $\left(\varphi_{a},\vartheta_{a}\right)$ and $\left(\varphi_{b},\vartheta_{b}\right)$

2.1 $$\begin{array}{r} \vec{a}(\varphi_{a},\vartheta_{a})=\left(\begin{array}{c} a_{z}\cdot \tan(\varphi_{a})\\ a_{z}\cdot \tan(\vartheta_{a})\\ a_{z} \end{array}\right)\;\text{with}\;a_{z}=\sqrt{\frac{1}{\tan^{2}(\varphi_{a})+\tan^{2}(\vartheta_{a})+1}}\\ \vec{b}(\varphi_{b},\vartheta_{b})=\left(\begin{array}{c} b_{z}\cdot \tan(\varphi_{b})\\ b_{z}\cdot \tan(\vartheta_{b})\\ b_{z} \end{array}\right)\;\text{with}\;b_{z}=\sqrt{\frac{1}{\tan^{2}(\varphi_{b})+\tan^{2}(\vartheta_{b})+1}}. \end{array}$$

For every incident direction, all diffracted directions must be considered. Since the DMD is basically a two-dimensional array of mirrors, we start with modelling a single mirror with defined dimensions

2.2 $$\vec{c}(s,t)=(s,t,0)\;\text{with}\;0\leq s\leq w,0\leq t\leq w.$$

Here, *w* is the width and height of the mirror, and *s* and *t* are parameters for the *x*- and *y*-directions, respectively. To model the tilt state each single mirror in a DMD array can be rotated by the angle γ around the normalized diagonal axis $\vec{n}=(n_{x},n_{y},n_{z})=\frac{1}{\sqrt{2}}(1,1,0)$ via the rotation matrix

2.3 $$\begin{array}{r} R_{n}(\gamma )=\left(\begin{array}{ccc} n_{x}^{2}(1-\cos\gamma )+\cos\gamma & n_{x}n_{y}(1-\cos\gamma )-n_{z}\sin\gamma & n_{x}n_{z}(1-\cos\gamma )+n_{y}\sin\gamma \\ n_{y}n_{x}(1-\cos\gamma )+n_{z}\sin\gamma & n_{y}^{2}(1-\cos\gamma )+\cos\gamma & n_{y}n_{z}(1-\cos\gamma )-n_{x}\sin\gamma \\ n_{z}n_{x}(1-\cos\gamma )-n_{y}\sin\gamma & n_{z}n_{y}(1-\cos\gamma )+n_{x}\sin\gamma & n_{z}^{2}(1-\cos\gamma )+cos\gamma \end{array}\right). \end{array}$$

To shift a single mirror, we use the native grid $\vec{m}(m_{x},m_{y})=m(m_{x},m_{y},0)$ with the micromirror pitch m=w+g. The gap between the non-tilted mirrors is *g*. $m_{x}\in N_{0}$ and $m_{y}\in N_{0}$ are the indices of the grid points (the single mirrors). This leads us to the following expression to describe each single mirror on the DMD:

2.4 $${\vec{d}}_{m_{x},m_{y}}(\gamma_{m_{x},m_{y}},s,t,m)=R(\gamma_{m_{x},m_{y}})\cdot \vec{c}(s,t)+m(m_{x},m_{y},0).$$

We can model monochromatic coherent light using the Fraunhofer/far-field approximation with a time-independent electric field, which depends on the beam profile $I_{0}(\vec{a},\vec{d})=|E_{0}(\vec{a},\vec{d}){|}^{2}$ projected onto the DMD

2.5 $$E(E_{0}(\vec{a},\vec{d}),\Phi )=E_{0}(\vec{a},\vec{d})\cdot e^{i\Phi }\;\text{with}\;\Phi =l\cdot \frac{2\pi }{\lambda }=\vec{d}(\vec{a}-\vec{b})\frac{2\pi }{\lambda }.$$

**Figure 2.**
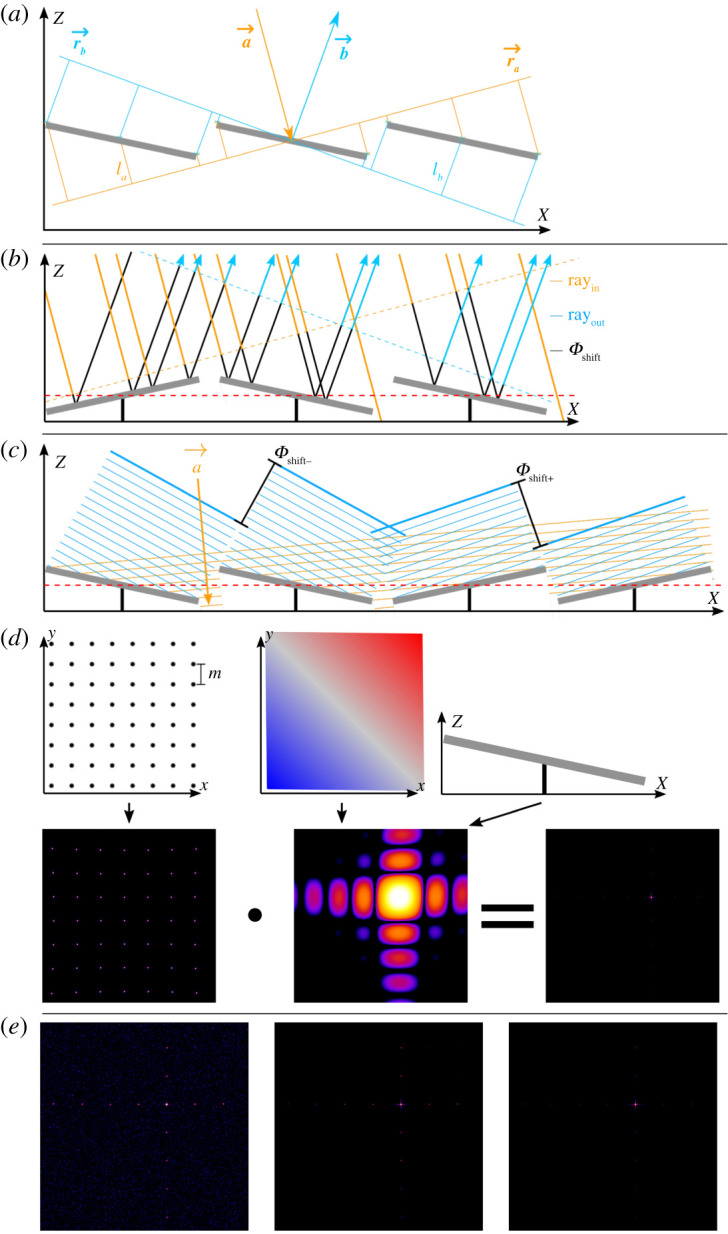
Illustration and comparison of algorithms for the simulation of diffraction with DMDs. Modelling of (coherent) light that is diffracted at a DMD surface. All diffraction images shown are in logarithmic intensity representation and were generated with an array of 50 × 50 micromirrors at 532 nm wavelength. Each micromirror has a size of 7.56 µm × 7.56 µm. $\varphi_{a}=-\vartheta_{\text{a}}=-21^{\circ }$ (blazed condition) was chosen as angle of incidence. The diffraction images are shown for both angles $\varphi_{b}$ and$\vartheta_{b}$ with an angle range of −15° to 15°. (*a*) General determination of the phase shift for different points of a planar wave front, which is incident on the DMD in direction $\vec{a}$ and is diffracted in direction $\vec{b}$. This approach is correct in the far-field/Fraunhofer approximation. (*b*) Ray tracing approach: Modelling of rays and their phase shift, which are incident on the DMD in the direction $\vec{a}$ and diffracted in the direction $\vec{b}$. (*c*) Analytical phase shift approach: Simplified modelling of diffraction images in the form of wavefronts reflected by micromirrors, where the diffraction image of a single mirror is analytically known. (*d*) Grating approach: In the upper row, the native grating of the DMD array, and a single mirror are shown schematically. In the lower row, the corresponding diffraction images and their product, which results in the diffraction image of the entire DMD, are shown. (*e*) Comparison of the diffraction images (from left to right): Ray tracing approach, analytical phase shift approach, grating approach. (Online version in colour.)

$E_{0}$ represents the incidence amplitude and Φ the resulting phase, which is dependent on the path length $l=l_{a}+l_{b}=\vec{d}\vec{a}-\vec{d}\vec{b}$ and the wavelength λ (figure 2*a*). For the electric field diffracted at a specific point on the DMD we obtain

2.6 $$E_{m_{x},m_{y}}^{\text{one}\text{point}}(\vec{a},\vec{b},\gamma_{m_{x},m_{y}},s,t,m,\lambda )=E_{m_{x},m_{y}}^{0}(\vec{a},\gamma_{m_{x},m_{y}},s,t)\cdot \exp\left(\frac{2\pi i}{\lambda }{\vec{d}}_{m_{x},m_{y}}(\gamma_{m_{x},m_{y}},s,t,m)\cdot (\vec{a}-\vec{b})\right).$$

To calculate the diffracted field distribution, we need to integrate over each single mirror and calculate the sum over the entire DMD with $N_{x}$ and $N_{y}$ as the number of single mirrors in *x*- and *y*-direction

2.7 $$\begin{array}{rl} & E_{\text{DMD}}(\vec{a},\vec{b},\gamma_{m_{x},m_{y}},w,m,N_{x},N_{y},\lambda )\\ & \;=\sum_{m_{x}=0,m_{y}=0}^{N_{x}-1,N_{y}-1}\int \int_{0}^{w}E_{m_{x},m_{y}}^{0}(\vec{a},\gamma_{m_{x},m_{y}},s,t)\cdot \exp\left(\frac{2\pi i}{\lambda }{\vec{d}}_{m_{x},m_{y}}(\gamma_{m_{x},m_{y}},s,t,m)\cdot (\vec{a}-\vec{b})\right)ds\;dt. \end{array}$$

This expression depends on many parameters and generally cannot be simplified further analytically. In order to be able to calculate diffraction patterns numerically and in adequate time, it is thus necessary to make further assumptions to simplify the calculation. Depending on how these assumptions are chosen, different approaches emerge which are capable of modelling different system constraints.

### (b) Ray tracing approach

We approximate integrating over every single mirror and summing over the DMD (equation (2.7)) by running a Monte Carlo simulation with rays which will be summed up (figure 2*b*). This yields a computationally feasible (if somewhat slow) simulation that does not introduce further constraints or approximations not inherent to the Monte Carlo process. To simulate a collimated Gaussian incident laser beam, we use a Gaussian probability density for the generation of *K* randomized incident beams with this equation of a line

2.8 $${\vec{f}}_{k}={\vec{h}}_{k}+u\vec{a}\;\text{with}\;k\in {\text{N}}_{0},u\in R\;\;\text{and}\;\;k<K,$$

and with ${\vec{h}}_{k}$ as support vector, $\vec{a}$ as normalized incident direction vector and the parameter *u*. Here, ${\vec{h}}_{k}\cdot \vec{a}=0$ because ${\vec{h}}_{k}$ and $\vec{a}$ are perpendicular to each other. ${\vec{h}}_{k}$ is Gaussian randomized for each ray ${\vec{f}}_{k}$. To obtain the point of diffraction on the DMD, we must calculate the intersection point ${\vec{p}}_{k}=(m_{x},m_{y},s,t,u)$ of ${\vec{f}}_{k}$ and $\vec{d}$. For each ray, we assume that the intensity is $E_{m_{x},m_{y}}^{0}=1$. This simplifies equation (2.6) to

2.9 $$E_{m_{x},m_{y}}^{\text{one ray}}(\vec{a},\vec{b},{\vec{p}}_{k},\lambda )=E_{in}(\vec{a},{\vec{p}}_{k},\lambda )\cdot E_{\text{out}}(\vec{b},{\vec{p}}_{k},\lambda )=\exp\left(\frac{2\pi i}{\lambda }\vec{d}({\vec{p}}_{k})\vec{a}\right)\cdot \exp\left(-\frac{2\pi i}{\lambda }\vec{d}({\vec{p}}_{k})\vec{b}\right).$$

A Gaussian beam profile is already considered by the probability distribution of the rays. The field composed of contributions from all rays needs to be summed up to result in the final ray tracing expression for the diffracted field distribution (figure 2*e*, left). This simplifies equation (2.7) to

2.10 $$E_{DMD}(\vec{a},\vec{b},\gamma_{m_{x},m_{y}},w,m,N_{x},N_{y},\lambda )=\sum_{k=0}^{K-1}\exp\left(\frac{2\pi i}{\lambda }\vec{d}({\vec{p}}_{k})(\vec{a}-\vec{b})\right).$$

In this ray-tracing approach, both the assumption of a Gaussian beam profile and especially of fully monochromatic (i.e. coherent) light could easily be changed. Thus, by using a different ray distribution, and by allowing for the single rays (equation (2.9)) to follow a wavelength and phase distribution when being summed up (equation (2.10)), it would easily be possible to simulate for example an arbitrary profile of a partially coherent source.

### (c) Analytic phase shifting approach

For this approach, we assume that the field amplitude over each single mirror is constant $E_{m_{x},m_{y}}^{0}(\vec{a},\gamma_{m_{x},m_{y}},s,t)\to E_{m_{x},m_{y}}^{0}(\vec{a},\gamma_{m_{x},m_{y}})$ and each single mirror can only be in the tilt state $\gamma^{-}$ or $\gamma^{+}$ (as reasonable assumption for the steady state of a DMD). A Gaussian beam profile can still be approximated by varying $E_{m_{x},m_{y}}^{0}$ over the different single mirrors, and as typically a reasonably sized array of mirrors is illuminated, the error introduced by this approximation is small. This provides us with the opportunity to solve the integral in equation (2.7) over a single mirror for $\gamma^{-}$ and $\gamma^{+}$, with the analytically known solution for $E_{\gamma^{\pm }}^{\text{single mirror}}(\vec{a},\vec{b},w,\lambda )$ (see dmd.nb in the attached repository). Instead of calculating the field for each mirror individually, we can use the fields of a reference mirror for $\gamma^{-}$ and $\gamma^{+}$ and apply the phase shift for the desired grid point $\left(m_{x},m_{y}\right)$ shown in figure 2*c*. This simplifies equation (2.7) for the diffracted field (figure 2*e*, middle) to

2.11 $$\begin{array}{rl} & E_{\text{DMD}}(\vec{a},\vec{b},\gamma_{m_{x},m_{y}},w,m,N_{x},N_{y},\lambda )\\ & \;=\sum_{m_{x}=0,m_{y}=0}^{N_{x}-1,N_{y}-1}E_{m_{x},m_{y}}^{0}(\vec{a},\gamma_{m_{x},m_{y}})\cdot E^{\text{single mirror}}(\vec{a},\vec{b},\gamma_{m_{x},m_{y}}^{\pm },w,\lambda )\cdot E_{m_{x},m_{y}}^{\text{shift}}(\vec{a},\vec{b},m,\lambda ). \end{array}$$

This approach is computationally much simpler, while only introducing a minor approximation. It was thus used for the design of our compact DMD-based SIM system [14].

### (d) Grating approach

Additionally to the assumptions for the analytic phase shifting approach, we assume here that the field amplitude over the entire DMD is constant ($E_{m_{x},m_{y}}^{0}(\vec{a},\gamma_{m_{x},m_{y}})=1$) and that all single mirrors are in the $\gamma^{-}$ or $\gamma^{+}$ state. The field $E_{\gamma^{\pm }}^{\text{single mirror}}(\vec{a},\vec{b},w,\lambda )$ can be considered as an envelope over the diffracted native DMD grid $E^{\text{grating}}(\vec{a},\vec{b},m,N_{x},N_{y},\lambda )$. This gives the opportunity to write equation (2.7) as follows:

2.12 $$E_{\text{DMD}}(\vec{a},\vec{b},\gamma^{\pm },w,m,N_{x},N_{y},\lambda )=E^{\text{single mirror}}(\vec{a},\vec{b},\gamma^{\pm },w,\lambda )\cdot E^{\text{grating}}(\vec{a},\vec{b},m,N_{x},N_{y},\lambda ).$$

To get the intensity $I_{\text{DMD}},$ we calculate and multiply both diffraction patterns $I_{\text{envelope}}$ and $I_{\text{grating}}$ (figure 2*d*), very similar to the case of the Young double slit experiment

2.13 $$I_{\text{DMD}}=|E_{\text{DMD}}{|}^{2}=|E^{\text{single}\;\text{mirror}}{|}^{2}\cdot |E^{\text{grating}}{|}^{2}=I_{\text{envelope}}\cdot I_{\text{grating}}.$$

Of course, this approach is constrained to the simulation of a DMD without a pattern being displayed. However, if only the diffractive nature of the DMD itself is of concern, this offers a computationally very effective solution.

### (e) Blaze condition approach

**Figure 3.**
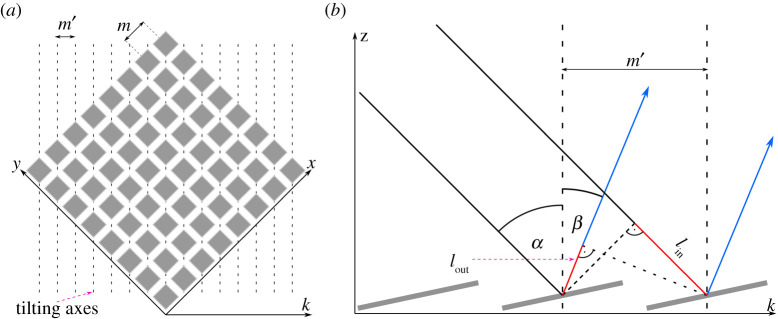
Blazed condition approach. (*a*) Schematic illustration of a DMD rotated by 45° around the z-axis. Furthermore, the tilt axes of the micromirrors, the grid constants *m* and *m'* and the coordinate axis *k* are shown. (*b*) Graphical representation of the calculation of the phase shift for light, which is incident along the KZ-plane on a DMD. (Online version in colour.)

We assume that the incident beam is perpendicular to the tilting axes $\vec{n}$ of the single mirrors. This leads to $\vec{a}=(a_{x},-a_{x},a_{z})$ and $-\varphi_{a}=\vartheta_{a}$. To imagine it simply we rotate the DMD by $45^{\circ }$ (figure 3*a*). To account for the rotation, we switch to new angle coordinates (figure 3*b*):

2.14 $$\alpha =\arctan\left(\sqrt{2}\cdot \text{tan}(\varphi_{a})\right);\beta =\arctan\left(\sqrt{2}\cdot \text{tan}(\varphi_{b})\right).$$

As new coordinate axes, we define $\vec{k}=(1,-1,0)$. Along $\vec{k}$ we get a new lattice constant $m^{\prime }=(1/\sqrt{2})m$.

The pathlength is given by

2.15 $$l=l_{a}-l_{b}=m^{\prime }(\sin(\alpha )-\sin(-\beta )).$$

For perfect constructive grating interference, the path difference *l* must be an integer multiple of the wavelength λ

2.16 $$n\lambda =m^{\prime }(\sin(\alpha )+\sin(\beta )).$$

If we have perfect constructive interference in the centre of the envelope, the blaze condition is fulfilled. The centre of the envelope can then be assumed as a reflection of the light incident on the surface of a single mirror and we define this as our diffraction angle β=−α+2γ. For this reflected envelope, we can now calculate the corresponding diffraction order

2.17 $$n=\frac{m^{\prime }}{\lambda }(\sin(\alpha )+\sin(-\alpha +2\gamma )).$$

If *n* is an integer the blaze condition is fulfilled. We use *v* to visualize this relationship

2.18 $$v=|\sin(n\pi )|=\left|\sin\left(\frac{\pi m^{\prime }}{\lambda }(\sin(\alpha )+\sin(-\alpha +2\gamma ))\right)\right|.$$

Here, *v*_0_, whenever the blaze condition is fulfilled. The choice of |sin⁡(nπ)| leads to a symmetric (negative and positive deviations contribute equally) and slightly nonlinear (small deviations contribute stronger) metric, which is well suited to visualize the blaze condition for different wavelengths λ and different incident angles α along the diagonal of the DMD (figure 3*b*).

While this approach is even more constrained, it has an important real-world application: it allows us to directly calculate incident and reflective angles when placing a DMD rotated by 45° on an optical table, where collimated beams are expected to run parallel to the table's surface. Here, it provides a quick solution for the instrument design. A similar approach was pursued by Li *et al*. [20].

## 3. Results and discussion

Each of the algorithms described above has certain advantages and disadvantages, which means that each algorithm has its own area of application. As shown in figure 1*e*, the ray-tracing approach, the analytic phase shifting approach and the lattice approach each deliver qualitatively the same results under identical boundary conditions. The positions of the individual diffraction orders are identical in the three algorithms. All diffraction images shown in this article were simulated with m=7.56 μm micromirror pitch and $\gamma^{\pm }=\pm 12^{\circ }$ as tilt angle, which corresponds to the dimensions of the *DLP® LightCrafter™ 6500* (*Texas Instruments)* [26] and some other DMDs. To obtain a clear visualization of the diffraction orders with large visible spots (Fourier broadening), the simulation is limited to an array of 50 × 50 mirrors being illuminated. Of course, when no or a different visualization is needed, the algorithms can be run for larger mirror arrays. Especially, when a small subset of possible angles is computed with a high sampling rate, a larger number of mirrors can and should be used, up to the full DMD size.

**Figure 4.**
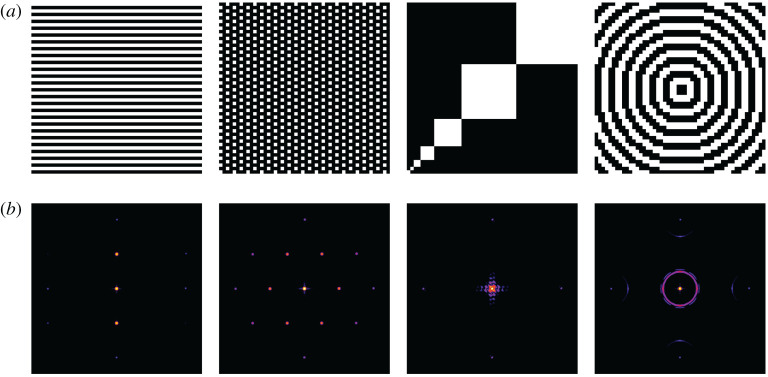
Comparison of diffraction images by different patterns generated on the DMD. (*a*) Different DMD patterns. From left to right: Horizontal lines; MAP-SIM example; MAP-SIM calibration pattern [30–32]; concentric circles. (*b*) Diffraction images of the DMD patterns shown in (*a*), simulated with the analytical phase shift approach with 50 × 50 micromirrors at 532 nm wavelength. For the angles of incidence $\varphi_{a}=-\vartheta_{\text{a}}=-21^{\circ }$ (blaze condition) was chosen. The diffraction images are shown for a range of −1.8° to 8.2° for $\varphi_{b}$ (*x* axis) and −8.2° to 1.8° for $\vartheta_{b}$ (*y* axis). The intensity distribution is shown on a logarithmic scale. (Online version in colour.)

The ray tracing approach is most flexible because it requires the least simplifying approximations. It can be used to calculate the diffraction patterns for any DMD-pattern. In addition, the approach could be extended relatively easily for arbitrary exposure beam profiles and incoherent light by specifying a corresponding spatial distribution and wavelength distribution and/or phase distribution for the random rays. Also, mechanical deformation of the DMD, i.e. non-flat surface structured, could be integrated into the model, and aid in determining their influence on the diffraction and thus illumination properties of the device. The main disadvantage of the ray tracing approach is that it is by far the slowest of the presented algorithms. Therefore, this algorithm is especially suitable for the simulation of incoherent or partially coherent light. The analytical phase shifter has the only disadvantage that, when compared with the ray tracing approach, it cannot be readily modified for use with incoherent illumination, or if done so, it would lose its speed advantage. Also, the granularity with which illumination intensity and mechanical deformations (which would be represented as phase shifts) could easily be simulated is limited to a single intensity and offset per mirror, introducing an approximation. For the illumination, this effect is not noticeable in practice. As its main advantage, the algorithm works much faster and is especially suitable for the calculation of diffraction images where the DMD is provided with a pattern (figure 4).

**Figure 5.**
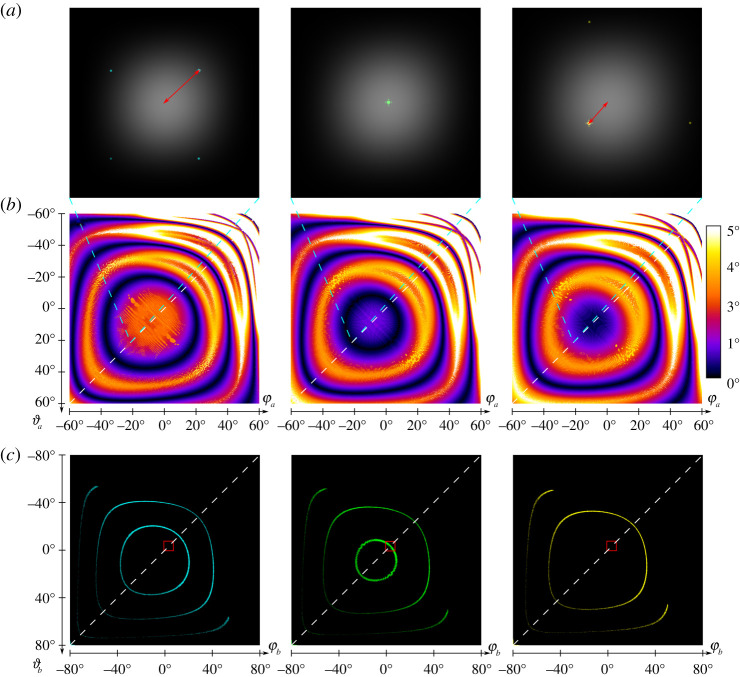
Analysis of the displacement between the envelope and the brightest diffraction order of the DMD. The columns from left to right correspond to the wavelengths 488 nm, 532 nm and 561 nm. Row (*a*) illustrates the displacement between the centre of the envelope (grey, linear intensity representation) and the brightest diffraction order of the native DMD grating (cyan, green, yellow, logarithmic intensity representation) using red arrows. The field of view ranges from −1° to 7° for $\varphi_{b}$ and −7° bis 1° for $\vartheta_{b}$ with a sampling rate of $0.1^{\circ }$ at an angle of incidence of $\varphi_{a}=-\vartheta_{\text{a}}=-21^{\circ }$. Row (b) visualizes the offset which can be determined from (*a*) for the angles of incidence $\varphi_{a}$ and $\vartheta_{a}$ ranging from −60° to 60° with a sampling rate of 0.2°. The dark areas with a displacement close to 0° indicate angles which fulfil a blaze condition and are well suited for illumination with coherent light. The cyan dotted lines between (*a*) and (*b*) indicate an angle of incidence of $\varphi_{a}=-\vartheta_{\text{a}}=-21^{\circ }$ for the diffraction images seen in (*a*). Row (*c*) shows the areas in the diffraction space of $\varphi_{b}$ and $\vartheta_{b}$ from −80° to 80°, each, in which the displacement between the centre of the envelope and the brightest diffraction order of the DMD is not more than 0.1°. Therefore, angles of incidence in a range of −60° to 60° used in (*b*) were used for $\varphi_{a}$ and $\vartheta_{a}$. The red boxes mark the diffraction space shown in (*a*). The white dotted lines in (*b*) and (*c*) correspond to the diagonal angles of incidence that are considered in the blazed condition approach. (Online version in colour.)

The grating approach is not able to simulate any patterns displayed on the DMD. It assumes that all mirrors are tilted in the same direction. Due to its high speed, however, the algorithm is very well suited for investigating the effects of different boundary conditions such as changes in wavelength and angle of incidence on the diffraction image generated natively by the structure of the micromirror array (figure 5). It can be used to determine the distance between the centre of the envelope and the brightest native grating order (figure 5*a*). If this distance is 0°, the blaze condition (Littrow configuration) is fulfilled. For the use of coherent light in e.g. a SIM microscope, this is exactly what is desired, because then an isotropic envelope field and intensity distribution is present in the Fourier plane. This has the consequence that the disadvantages of the blazed grating effect of DMDs can be negated by exploiting the blaze condition. This is true for a range of angles of incidence which are shown in black in figure 5*b*. These angles of incidence that fulfil the blaze condition then result in the angles of diffraction marked in figure 5*c*.

If a DMD-based SIM microscope aims to provide multiple excitation wavelengths that share a single DMD, then it is necessary that for each wavelength the blaze condition is fulfilled. A possible approach to solving this problem is to use the $\gamma^{-}$ position of the micromirrors for one wavelength and the $\gamma^{+}$ position for the other wavelength. As shown for example for 488 nm or 638 nm in combination with 561 nm in figure 7*a*, there are two configurations in which the blaze condition is fulfilled for both wavelengths. This choice of wavelengths is desirable, as arguably 488 nm, 561 nm and 638 nm (all of course allowing for some variation) are very compatible with typical dyes used in fluorescence microscopy. This configuration is certainly possible but somewhat complex to achieve experimentally (as multiple independent tip/tilt axis have to be precisely aligned) and it is also very susceptible to the slightest change in angle of the micromirrors (we found e.g. 0.3° in [14]). Brown *et al*. were able to construct such a system [22] offering three excitation wavelengths (they chose a triplet of 473 nm, 532 nm and 635 nm). They use a (semi-)analytic approach to model the blazed grating effect, developed independently of the approaches presented here, while providing compatible results, which we view as an important cross-check. Their experimental realization, including a voice-coil mirror for the dynamic switching between incidence angles, is very functional but also highlights the complexity it introduces.

**Figure 6.**
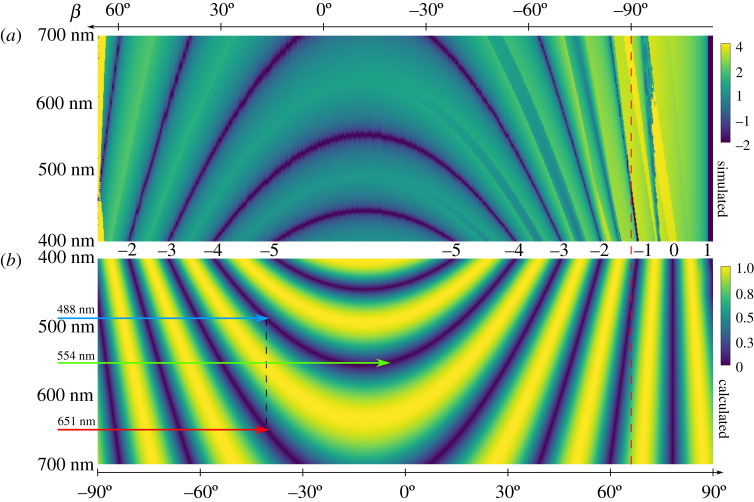
Blaze condition along the diagonals shown in figure 3*b*. Visualization of the blaze condition along the diagonals shown in figure 3*b* for the visible spectrum. In the dark areas the blaze condition can be considered fulfilled. For orientation, the wavelength range is shown on the left-hand side. The angles of incidence and diffraction are shown at the top and the bottom of the graph. The vertical red dashed line marks the diffraction angle of 90°. Angles above 90° are irrelevant for practical applications. (*a*) The blaze condition is measured by the distance between the centre of the envelope and the brightest diffraction order of the DMD, which was simulated by the grating approach. The measured distances in degrees are shown as logarithmic intensity distribution. (*b*) Visualization v=|sin⁡(nπ)| of the blaze condition using the blazed-grating approach. The integer diffraction orders belonging to the blaze conditions are shown in the middle between (*a*) and (*b*). The blue, red and green arrows point to an exemplary three-colour combination for a DMD-SIM microscope with 488 nm, 651 nm and 554 nm. The black vertical dotted line is intended to illustrate that 488 nm and 651 nm fall on the DMD at the same angle of incidence. See also [20] for a visualization in similar style, obtained by Li *et al*. with their DMD modelling approach. (Online version in colour.)

For a multi-colour implementation of a cost-effective DMD SIM[14], we aim to reduce opto-mechanical complexity, ideally avoiding dynamic opto-mechanical components or two-dimensional alignment (non-horizontal beam paths). Therefore, we propose to limit the angle of incidence and thus also the outgoing angle for the optical axis of the DMD-SIM microscope to the diagonal perpendicular to the tilting axis of the micromirrors. This corresponds to a rotation of the DMD of 45° around the *z*-axis (figure 3). This limits the possible angles of incidence as well as the angles of diffraction for the optical axis to the white dotted diagonal in figure 5*b*. This reduces the number of degrees of freedom of the incident laser beam $(\varphi_{a},\vartheta_{a})\to \alpha$ and the optical axis $(\varphi_{b},\vartheta_{b})\to \beta$ from two to one each, as described in equation (2.14). The experimental implementation thus becomes much easier, because the exposure path of the microscope can be set up parallel to the optical table, as usual. Now, however, the wavelengths must be chosen skilfully. For a more precise analysis of the blaze condition along the diagonal, both the grating approach and the blazed condition approach are suitable (figure 6).

**Figure 7.**
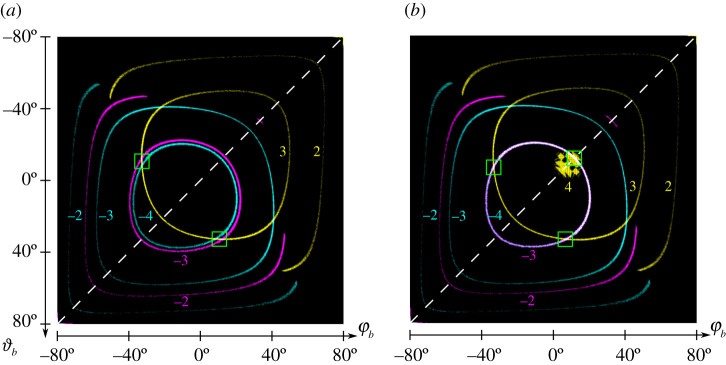
Multicolour diffraction angles with blaze condition. Areas in the diffraction space for $\varphi_{b}$ and $\vartheta_{b}$ of −80° to 80°, each, in which the displacement between the centre of the envelope and the brightest diffraction order of the DMD is not more than 0.1°. Therefore, angles of incidence in a range of −60° to 60° were used for $\varphi_{a}$ and $\vartheta_{a}$. The white dotted lines correspond to the diagonal angles of incidence that are considered in the blazed condition approach. The coloured numbers near the ring structures represent the corresponding diffraction orders. (*a*) Typical wavelengths 488 nm (displayed in cyan) & 638 nm (displayed in magenta) with $\gamma^{-}=-12^{\circ }$ and 561 nm (displayed in yellow) with $\gamma^{+}=12^{\circ }$ are not well suited for multicolour applications. The green boxes mark possible configurations for two colour configurations of 488 nm and 561 nm or 638 nm & 561 nm. (*b*) Optimized multicolour wavelengths 488 nm (cyan) and 651 nm (magenta) with $\gamma^{-}=-12^{\circ }$ and 554 nm (yellow) with $\gamma^{+}=12^{\circ }$. The green boxes mark possible configurations for three colour configurations. (Online version in colour.)

Both approaches show qualitatively the same results, therefore, it is sufficient to refer to the blaze condition approach in the following. It is possible to use two wavelengths $\lambda_{1}$ and $\lambda_{2}$ with the same angle of incidence $\alpha_{1,2}$ and diffraction β with the same tilt state $\gamma^{-}=-12^{\circ }$. Starting from equation (2.16), it follows that

3.1 $$n_{1}\lambda_{1}=n_{2}\lambda_{2}.$$

As integer diffraction orders $n_{1}=-4$ and $n_{2}=-3$ are suitable for the visible range. If we pretend $\lambda_{1}=488\;\text{nm}$, we obtain

3.2 $$\lambda_{2}=\frac{n_{1}}{n_{2}}\cdot \lambda_{1}=650.67\;\text{nm}\;\text{with}\;\alpha_{1,2}=-40.6^{\circ }\;\;\text{and}\;\;\beta =16.6^{\circ }.$$

If a third excitation colour is supposed to use the other tilt state $\gamma^{+}=12^{\circ }$, we get $\alpha_{3}=-\beta +2\gamma^{+}=7,4^{\circ }$ as incidence angle and must assume $n_{3}=4$ to be in the visible range, which then limits the wavelength to

3.3 $$\lambda_{3}=\frac{m^{\prime }}{n_{3}}(\text{sin}(\alpha_{3})+\sin(\beta ))=553.93\;\text{nm}\text{.}$$

This combination of these three wavelengths, arranged at $\left(-40.6^{\circ },488\;\text{nm}\right)$, $\left(-40.6^{\circ },651\;\text{nm}\right)$ and $\left(7.4^{\circ },554\;\text{nm}\right)$ is indicated in figure 6 as a possible configuration for a three-colour DMD-SIM microscope. Given that these wavelengths are close to the typical combinations of blue (472 nm to 488 nm), yellow (561 nm to 568 nm) and red (630 nm to 650 nm) laser excitation sources used in fluorescence microscopy, and that suitable diode laser sources (even offering temperature-based fine-tuning of their emission wavelength) are readily available, this combination should make for a surprisingly capable three-colour DMD-based microscope.

Further possible configurations that closely match typical excitation wavelength are triples of e.g. 473 nm,631 nm and 551 nm or 491 nm,655 nm and 555 nm. It is important to note here that $\lambda_{3}$ depends very much on the tilt angles of the micromirrors, and thus might have to be adjusted based on the parameters of the specific DMD device. For $\lambda_{1}$ and $\lambda_{2}$_,_ the tilt angle is of less importance. Looking at the diffraction space in which the blaze condition is fulfilled for typical wavelengths (figure 7*a*), it is noticeable that, using the same tilt state, no matches are found. The ring-like structures in figure 7 represent the different diffraction orders. Their size depends on wavelength and diffraction order and for clarity they are shown in different colours. As mentioned earlier, using the other tilt state results in matches, which are not on the diagonal and therefore not suitable for experiments. But if we choose the wavelengths carefully for multicolour applications (figure 7*b*), the circular structure of the -4th and -3rd diffraction order of 488 nm and 651 nm using the same tilt state match exactly. If we use the other tilt state for 554 nm, there is, besides the matches outside the diagonal, another one on the diagonal, which is formed by a collapsing/emerging circular structure of the 4th diffraction order. This configuration, already described above, is therefore ideal for a three-colour DMD-SIM microscope.

## 4. Conclusion

The framework and the software tools provided here provide a rather universal set of simulation tools for experimentalists planning to use DMDs as SLMs with coherent light sources in SIM microscopes or similar applications. Compared to ferro-electric light modulators (FLCoS-SLM), DMDs are faster and more cost efficient, and they allow for a more compact design due to the smaller micromirror/pixel pitch. Also, the use of FLCoS-SLMs requires the implementation of a much more complicated timing scheme because of the limited duration during which a specific pattern can be projected by these devices. DMDs have no such constraints and can switch at speeds up to an order of magnitude faster than FLCoS-SLMs.

We have developed four different approaches to simulate coherent light diffracted and reflected by a DMD (table 1). The results provide practical design suggestions for circumventing the undesired blazed-grating effect of DMDs. Our simulation results agree with the independent findings of Brown *et al.*, who were able to successfully construct a multi-colour DMD SIM offering three excitation wavelengths [22]. Following our analysis of suitable wavelength, and building on top of an existing opto-mechanical platform [14], it should now also be possible to design a multicolour DMD-based SIM microscope with simple, passive opto-mechanics which can fully use all of the speed and cost advantages of a DMD mentioned before. Additionally, our simulation platform offers the flexibility to be extended, so that the use of partially coherent light sources and the effects of mechanically deformed DMDs can be explored. Especially for the latter, we hope that with a combination of inteferometric measurements and extensions of the ray tracer simulation, we can in the future explore how the optical quality (flatness, etc.) of the DMD influences illumination properties. We are currently building a two-colour DMD SIM microscope and plan to add a third colour to it in its final implementation.

**Table 1.** Comparison of all discussed approaches. This table gives a compact summary of the discussed approaches. The runtimes for the approaches were measured with 50 × 50 mirrors at a resolution of 1500 × 1500 pixels in diffraction space on an Intel Core i5 4690 4×3.50 GHz with a NVIDIA GeForce GTX1060 6GB. The ray tracing approach was measured with 100 000 rays. The runtimes can vary significantly depending on the approach, the number of mirrors, the resolution and the computer used.

	ray tracing	analytic phase shifting	grating	blaze condition
capable of patterns	+ yes	+ yes	− no	− no
speed (Time CPU/GPU)	– very slow (84 min/——)	− slow (7.5 min/3 sec)	+ fast (3.2 sec/1.2 sec)	++ instant
use case	incoherent light	specific DMD-patterns	native pattern under different conditions	analysis of the diagonal

The results shown in this article can easily be reproduced with the ImageJ/Fiji plugin which we provide as an open access download. The plugin contains all approaches and algorithms presented in this article, which can be adapted to different system conditions. For a more detailed insight into the plugin and some mathematical additions, we recommend a look at the electronic supplementary material.

Following the spirit of open science, we provide all source code and raw data for the results presented in this manuscript to the scientific community. All code is openly accessible under GPLv2 (or later) license and can be found in online repositories under github.com/fairSIM and github.com/biophotonics-bielefeld.

## Data accessibility

A software package to simulate with the introduced algorithms the diffraction patterns of DMDs and the introduced further analysis depending on the angle of incidence, light wavelength and DMD pattern has been created. The software is written in Java to work as a plugin to scientific image processing package ImageJ/FIJI [27,28] and provides GPU acceleration through the jCuda framework [29]. The source code is available under an open-source (GPLv3) licence in an online repository: github.com/biophotonics-bielefeld/coherent-dmd-sim-simulator.
